# Klotho‐mediated targeting of CCL2 suppresses the induction of colorectal cancer progression by stromal cell senescent microenvironments

**DOI:** 10.1002/1878-0261.12577

**Published:** 2019-10-06

**Authors:** Yangyang Liu, Jie Pan, Xia Pan, Lunpo Wu, Jun Bian, Zhenghua Lin, Meng Xue, Tingting Su, Sanchuan Lai, Fei Chen, Qiwei Ge, Luyi Chen, Shufang Ye, Yabi Zhu, Shujie Chen, Liangjing Wang

**Affiliations:** ^1^ Department of Gastroenterology Second Affiliated Hospital of Zhejiang University School of Medicine Hangzhou China; ^2^ Institution of Gastroenterology Zhejiang University Hangzhou China; ^3^ Department of Gastroenterology The Sixth Affiliated Hospital of Wenzhou Medical University Lishui People's Hospital China; ^4^ Department of Endocrinology and Metabolism Second Affiliated Hospital of Zhejiang University School of Medicine Hangzhou China; ^5^ Department of Gastroenterology Sir Run Run Shaw Hospital Zhejiang University Hangzhou China

**Keywords:** CCL2, colorectal cancer, Klotho, senescence

## Abstract

Senescent microenvironments play an important role in tumor progression. Here, we report that doxorubicin (DOX)‐pretreated or replicative senescent stromal cells (WI‐38 and HUVEC) promote colorectal cancer (CRC) cell growth and invasion *in vitro* and *in vivo*. These pro‐tumorigenic effects were attenuated by exogenous administration of Klotho, an anti‐aging factor. We subsequently identified several senescence‐associated secretory phenotype (SASP)‐associated genes, including CCL2, which were significantly upregulated in both types of senescent stromal cells during replication and DNA damage‐induced senescence. Importantly, we found that the secretion of CCL2 by senescent stromal cells was significantly higher than that seen in nonsenescent cells or in senescent cells pretreated with Klotho. Notably, CCL2 was found to accelerate CRC cell proliferation and invasion, while this effect could be blocked by administration of a specific CCR2 antagonist. We further show that Klotho can suppress NF‐κB activation during DOX‐induced senescence and thus block CCL2 transcription. Low expression of Klotho, or high expression of CCL2 in patient tumor tissues, correlated with poor overall survival of CRC patients. Collectively, our findings suggest that senescent stromal cells are linked to progression of CRC. Klotho can suppress the senescent stromal cell‐associated triggering of CRC progression by inhibiting the expression of SASP factors including CCL2. The identification of key SASP factors such as CCL2 may provide potential therapeutic targets for improving CRC therapy.

**Abbreviations**
CM: conditioned media
CRC: colorectal cancer
DMEM: Dulbecco's modified Eagle medium
DOX: doxorubicin
PDL: population doubling levels
SASP: senescence‐associated secretory phenotype

## 1 Introduction

Cellular senescence was first described by Hayflick *et al*. as a state of growth arrest (Campisi, 2013). In contrast to cancer cells, the proliferative capacity of normal cells is probably limited by replicative exhaustion. This senescence mechanism helps protect cells from malignant transformation. Although metabolically active, senescent cells generally do not respond to growth factors. Moreover, crucial tumor suppressors such as INK4A and ARF have also been shown to be highly expressed in senescent cells (Collado and Serrano, 2010; Courtois‐Cox *et al*., 2008), potentially limiting their excessive or aberrant cellular proliferation. Therefore, the induction of cellular senescence may represent a protective event limiting tumor progression.

The potential interplay between tumor cells and surrounding senescent stromal cells is largely unexplored. A series of studies have suggested that senescent cells can act as a barrier to cancer development, which has been termed ‘oncogene induced senescence (OIS)’ (Aird and Zhang, 2015). Accumulating evidence has suggested that cellular senescence is also involved in maintenance of the tumor microenvironment and helps protect the tumor cells from the stresses generated through antitumor treatment (Gordon and Nelson, 2012). Several studies have shown that senescent stromal cells can stimulate premalignant and malignant epithelial cells to grow *in vitro*, and to form experimental tumors in animal models (Agrawal *et al*., 2018; Bhowmick *et al*., 2004; Lee *et al*., 2017; Taddei *et al*., 2014; Wang *et al*., 2017). This phenomenon may be attributed to the secretion of bioactive substances by a senescence‐associated secretory phenotype (SASP) that is characterized by the production of various chemokines, proteases, growth factors, and inflammatory cytokines (Coppé *et al*., 2010). The secretory factors identified include matrix metalloproteinases, IL‐6, IL‐8, and TGF‐β, which can help remodel the microenvironment, alter epithelial differentiation, promote endothelial cell motility, and stimulate tumor cell growth (Lasry and Ben‐Neriah, 2015).

In the present study, we sought to better characterize the molecular alterations that occur during the process of stromal cell senescence and to further identify factors in the senescent microenvironments potentially linked to the neoplastic progression of colonic epithelium. To this end, two approaches were used to induce cellular senescence, namely replication and DNA damage‐mediated senescence. We subsequently identified a subset of SASPs that appear capable of affecting adjacent colonic epithelial growth. Using *in vitro* stromal/tumor cell co‐culture, and *in vivo* subcutaneous tumor formation models, we could further show that senescent stomal cells can promote the growth and invasion of experimental colorectal cancer (CRC). Importantly, these pro‐tumorigenic effects were attenuated by exogenous administration of Klotho, a classical anti‐aging protein (Kuro‐o *et al*., 1997; Kurosu *et al*., 2005) which we, and others, have previously shown to be tumor suppressive (Ibi *et al*., 2017; Ligumsky *et al*., 2015; Lojkin *et al*., 2015; Pan *et al*., 2011; Wang *et al*., 2011). We further demonstrate that Klotho can suppress the senescent stromal cell‐triggering of CRC progression by inhibiting CCL2 secretion, most likely through effects on the NF‐κB signaling pathway. Low expression of Klotho, or high expression of CCL2 proteins in tumor tissues, could be correlated with poor overall survival of CRC patients, suggesting that CCL2 represents a promising relevant biomarker and potential target for CRC.

## 2 Materials and methods

### 2.1 Cell lines

Colon cancer cell lines (RKO, LoVo) and human lung fibroblast cells (WI‐38) were obtained from American Type Culture Collection (ATCC, Manassas, VA, USA) and routinely cultured in Dulbecco's modified Eagle medium (DMEM) supplemented with 10% FBS and antibiotics (penicillin–streptomycin) at 37 °C in a 5% CO_2_ incubator. The human umbilical vein endothelial cell (HUVEC) was obtained from ATCC and maintained in CC‐3162 EGM‐2 BulletKit (Lonza, Walkersville, MD, USA), supplemented with 2% FBS, IGF‐1 0.5 mL, rh‐EGF 0.5 mL, rh‐FGF 2 mL, heparin 0.5 mL, hydrocortisone 0.2 mL, VEGF 0.5 mL, ascorbic acid 0.5 mL, and gentamicin sulfate 0.5 mL.

WI‐38 and HUVEC cells were cultured to a confluent state and counted. Population doubling levels (PDL) during each passage were calculated by the equation *A* = 3.32 (log*N*_2_ − log*N*_1_) + *X* (*A*, added population doubling level; *N*_2_, collected cell number at the end of each passage; *N*_1_, cell number at the beginning of each passage).

### 2.2 Induction of cell senescence

To induce replicative senescence, confluent WI‐38 and HUVEC cells were passaged until morphological signs of senescence emerged along with cessation of growth. For induction of senescence by doxorubicin (DOX; Sigma Aldrich, St. Louis, MO, USA), WI‐38 and HUVEC cells were cultured in the presence of doxorubicin (50 and 80 nm, respectively) in complete culture media for 72 h. Cells were then washed with PBS three times and left for at least 3 days prior to use. Senescence was documented by the senescence‐associated β‐galactosidase assay (SA‐β‐gal). SA‐β‐gal activity was measured using a standard protocol as described previously (Debacq‐Chainiaux *et al*., 2009). Briefly, cells were washed twice with PBS and fixed with fixative solution for 15 min at room temperature. The fixed cells were then incubated at 37 °C overnight without CO_2_ with X‐gal and supplements. Cells positive for blue staining were counted as senescent. To quantify SA‐β‐gal‐positive cells, cells were counted in three random fields and the percentage of positive cells was assessed. Due to the restricted replication potential of primary cells, we used parental WI‐38 and HUVEC cells between PDL 40–50 with less than 20% senescent cells as nonsenescent controls.

### 2.3 Treatment with Klotho and the collection of conditioned media

Human recombinant Klotho protein (R&D System, Minneapolis, MN, USA) was diluted with DMEM to a working solution of 100 ng·mL^−1^ prior to each experiment; 24 h after cell plating, WI‐38 and HUVEC cells were incubated with a final Klotho concentration of 10 ng·mL^−1^ for 48 h. To prepare the conditioned media (CM), senescent cells, nonsenescent cells, and cells with Klotho pretreatment were incubated with fresh medium for 72 h. The culture media was then harvested and centrifuged for 5 min. The supernatant was collected and stored at −80 °C before use.

### 2.4 Quantitative reverse transcription‐PCR

Total RNA (1 μg) was isolated using Trizol reagent (Invitrogen, Camarillo, CA, USA) following the manufacturer's instructions and reverse transcribed into cDNA with PrimeScript™ RT reagent Kit (Takara, Kusatsu, Japan). qPCR was then performed using SYBR Premix Ex Taq (Takara) on a LightCycler 480 (Roche, Mannheim, Germany) PCR instrument. Data were analyzed using the comparative *C*_t_ method ($2^{-\Delta \Delta C_{t}}$). Each sample was tested in triplicate. The steady‐state mRNA expression levels of a series of SASP‐associated genes previously reported (Coppé *et al*., 2010, Table S2) were evaluated. The relative values of DOX‐induced senescent cells to nonsenescent young cells were calculated to identify genes significantly up‐regulated in the DOX‐induced senescent cells. Cells pretreated with Klotho and then induced by DOX were also compared to DOX‐induced senescent cells to evaluate the effect of Klotho on senescent cells. From these calculations, an expression heatmap was generated using heatmap illustrator software (Heml software, Wuhan, Hubei, China).

### 2.5 Immunoblotting

Cells were harvested and extracted using lysis buffer (Tris/HCl, SDS, mercaptoethanol, and glycerol), supplemented with protease and phosphatase inhibitors (Selleck, Munich, Germany). PAGE was run according to a standard protocol and transferred onto polyvinylidene fluoride (PVDF) membranes (Merck Millipore, St. Louis, MO, USA). Membranes were blocked using 5% milk powder. Primary antibodies (Cell Signaling Technology, Beverly, MA, USA) directed against p53, p21, NF‐κB, p‐NF‐κB, I‐κB, and p‐I‐κB were used. The immunoreactive bands were visualized using an ECL Plus Kit according to the manufacturer's instructions. The relative protein levels in the various cell preparations were normalized to the level of β‐actin or GAPDH protein present.

### 2.6 Cell viability and colony formation assays

Cell viability was assessed using the Cell Counting Kit‐8 (CCK‐8; Dojin Laboratories, Kumamoto, Japan) applied according to the manufacturer's protocol. Colon cells were seeded in flat bottom 96‐well plates at a density of 3–4 × 10^3^ cells/well. Cells were then treated with the different indicated CM for 24, 48, 72, 96, or 120 h. Absorbance was measured at 450 nm after 2‐h incubation with CCK‐8 assay solution.

For the colony formation assay, cells were seeded in a 6‐well culture plate and incubated for 14 days. Colonies were counted only if they contained more than 50 cells, and the cell colonies were stained with 0.1% crystal violet.

### 2.7 Cell migration and invasion assay

Cell migration was assessed using a modified Boyden transwell chamber assay (Coring, NY, USA). Briefly, colon cells were plated in the upper chamber in 200 μL serum‐free medium, while the lower chamber was filled with 600 μL of conditioned medium as a chemoattractant. After 20‐h incubation, migrated cells were stained with DAPI staining solution. Cell numbers were randomly counted in three randomly chosen fields.

For invasion assays, 24‐well membrane covered inserts (Corning, NY, USA) were coated with 60 μL of BD Matrigel at 37 °C for 6 h. Harvested colon cancer cells were added to the upper compartment and conditioned medium as a source of chemoattractant was placed in the bottom of the chamber. The medium was removed from the upper chamber after 30 h. Invaded cells were fixed with 100% methanol and stained with 0.1% crystal violet. The number of migrated cells was then counted in three randomized fields.

### 2.8 Subcutaneous xenograft mice

For tumorigenicity assay, colon cancer cell LoVo (2 million cells) and fibroblasts (1 million cells) prepared with different states of senescence were subcutaneously injected into the left dorsal flank of 4‐week‐old male nude (nu/nu) mice (*n* = 6 each group). Palpable tumor size was measured every 2 days for 2–3 weeks using a digital caliper. Tumor volume (*V*) was estimated by measuring the longest diameter (*L*) and shortest diameter (*W*) of the tumor and calculated by the formula *V* = 0.5 × *L* × *W* ^2^. All experimental procedures were approved by the Animal Ethics Committee of Zhejiang University.

### 2.9 ELISA assay

The supernatants of WI‐38 and HUVECs were collected after treatment as indicated. Human CCL2 levels were measured using an ELISA Kit (R&D Systems) following the manufacturer's instructions and quantified using a microtiter plate reader at 450 nm wavelength.

### 2.10 Luciferase reporter activity analysis

The dual‐luciferase reporter assay system (Promega, Madison, WI, USA) was used to measure NF‐κB‐driven reporter gene activity. Cells were transfected in 24‐well plates with 6 ng Renilla luciferase control vector and 500 ng of the indicated plasmids, with the reporter gene plasmid in the presence of 2 μL FugeneHD (Promega). Firefly luciferase activity was measured with dual‐luciferase Reporter Assays (Promega) and normalized to Renilla luciferase activity levels. Luminescence was measured with a GloMax™ 20/20n luminometer (Promega).

### 2.11 Immunohistochemistry

Formalin‐fixed, paraffin‐embedded specimens (143 samples) were obtained from Sir Run Run Hospital, Zhejiang University, from January 2011 to June 2013. After informed written consent was obtained, patients who received surgical resection of primary cancer, but had no prior chemotherapy, radiotherapy, or other treatments before surgery were included in the study cohort. Clinicopathological features including age, gender, pathology, differentiation, TNM staging, lymph node metastasis, and follow‐up data were recorded. The grade and clinical staging of tumors were based on the American Cancer Committee (AJCC) guideline. The study methodologies conformed to the standards set by the Declaration of Helsinki and were approved by the local ethics committee.

Tissue sections were incubated in 3% hydrogen peroxide for 10 min at room temperature and then in blocking serum (10% normal goat serum in PBS) for 1 h. Primary rabbit antibody (Klotho; Abcam, Cambridge, UK) incubation was carried out at 4 °C overnight. Sections were then washed and incubated with secondary antibody for 1 h. DAB incubation was applied on to the sections before counterstaining with hematoxylin for 1 min. Images were obtained using a Leica DMRB microscope (Leica Microsystems, Wetzlar, Germany).

The expression of Klotho IHC score was calculated based on the extent (0–4 score) and the intensity (0–3 score) of staining, ranging from 0 to 12 (Qian *et al*., 2017). The expression of Klotho was then divided into 2 groups according to IHC score (high group 5–12 and low group 0–4). Each sample was scored independently by two pathologists, and the average of the two values was taken.

### 2.12 Statistical analysis

All statistical analyses were performed using graphpad prism, version 7.0 (GraphPad Software, San Diego, CA, USA) or spss, version 23.0 (SPSS Inc, Chicago, IL, USA). All assays were performed in triplicate, and results were expressed as mean ± standard deviation SD (continuous variables) or described as frequency and percentage (categorical data). A 2‐tailed, unpaired, or paired Student's *t* test was performed to compare the variables of two groups, and ANOVA test was used for multigroup comparisons. Overall survival was evaluated by the Kaplan–Meier survival analysis. COX proportion hazard regression model was performed to assess the prognostic value of Klotho expression. Differences were considered statistically significant when *P *<* *0.05.

## 3 Results

### 3.1 Senescent stromal cells promote colon cancer cells growth and invasion

**Figure 1:**
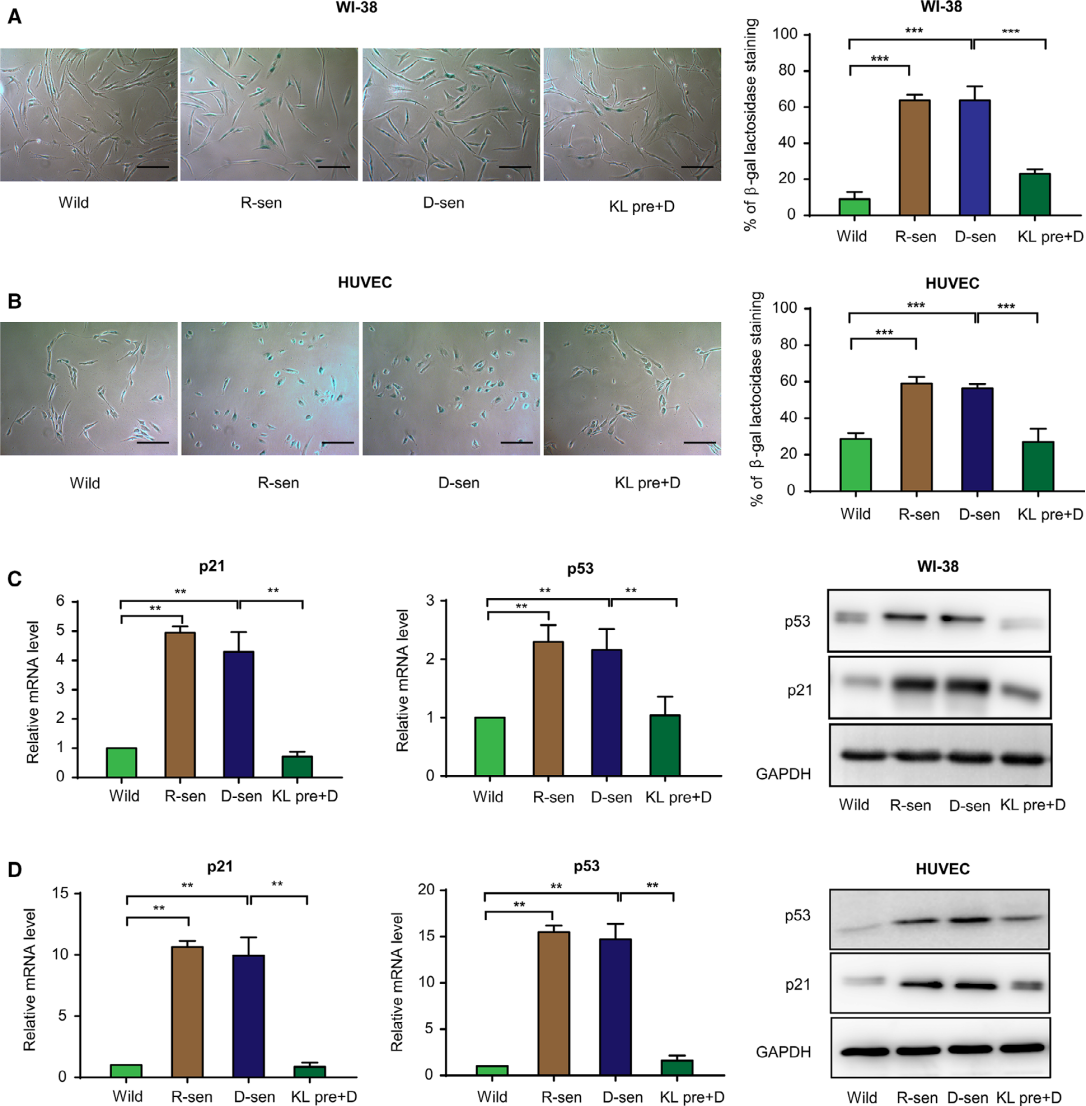
Klotho inhibits DOX‐induced senescence in stromal cells. Senescence‐associated β‐galactosidase staining of WI‐38 cells (A) and HUVEC cells (B) with wild‐type, replicative senescence (R‐sen), DOX‐induced senescence (D‐sen), and Klotho pretreatment (KLpre+D) are shown. Scale bar: 400 μm, 10× magnification. The percentage of SA‐β‐gal‐positive cells was evaluated for each group and showed that pretreatment with Klotho inhibited the senescence induced by replication or DOX. The results from three independent experiments are presented as mean ± SD. Relative mRNA and protein levels of p21 and p53 with indicated treatment for WI‐38 cells (C) and HUVEC cells (D) are shown. Induction of senescence increased expression of p21 and p53, which was attenuated by Klotho pretreatment in both cell lines. GAPDH was used as an internal control. Error bars are represented as mean ± SD (*n* = 3). *P*‐values was analyzed with one‐way ANOVA. **P <* *0.01; ***P <* *0.001.

**Figure 2:**
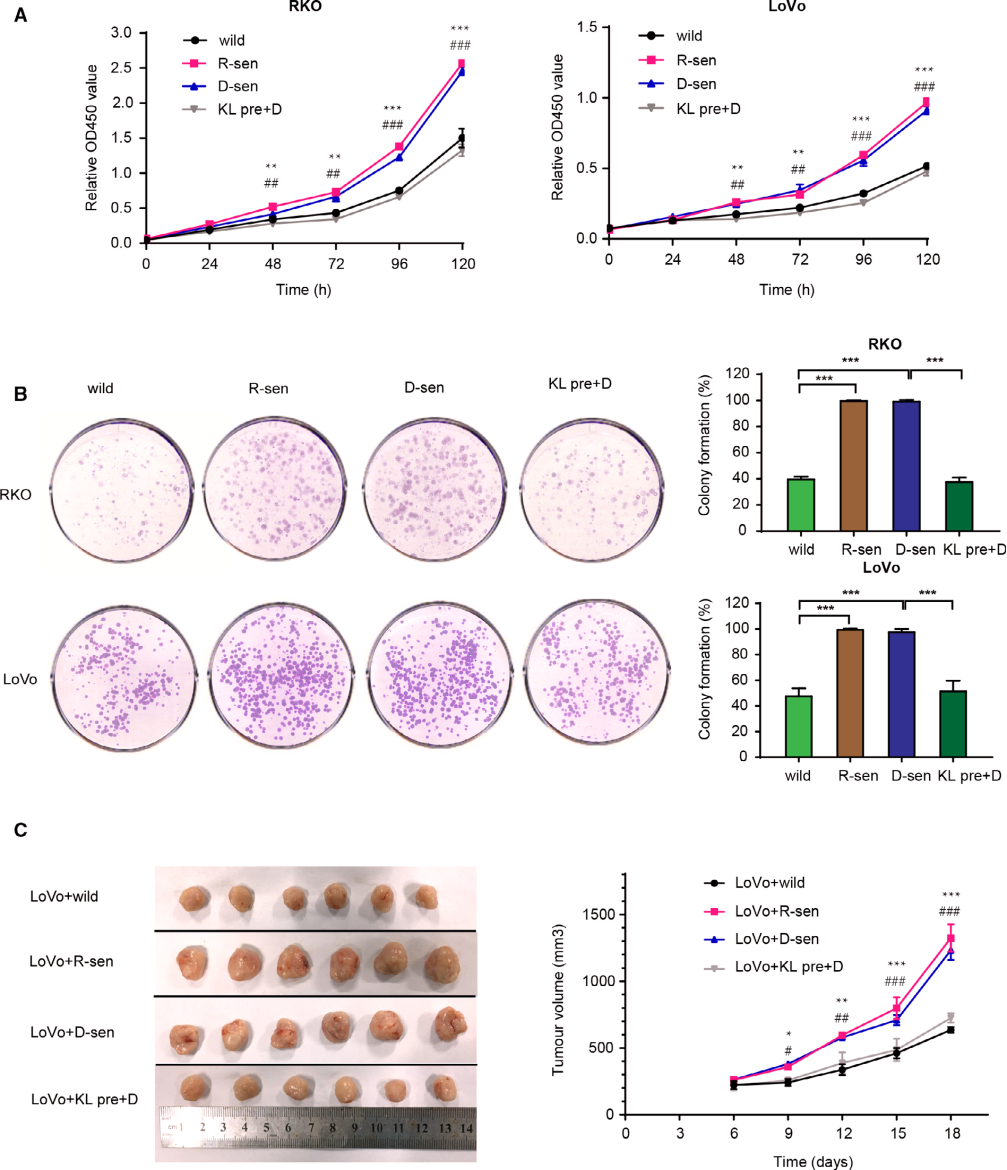
The effects of senescent fibroblasts and Klotho on CRC cell growth. (A) Cell viability was assayed for the RKO and LoVo cell lines and showed that CM from replicative (R‐sen) or DOX‐induced (D‐sen) senescent WI‐38 cells could promote CRC cell line proliferation, while pretreatment with Klotho (KL pre+D) inhibited the CRC cell growth (*n* = 3). Similar results were obtained from a colony formation assay for both cell lines (B) (*n* = 3). (C) LoVo cells were subcutaneously injected into the flank of nude mice and grew to a larger tumor size when co‐implanted with replicative (R‐sen) or DOX‐induced (D‐sen) senescent WI‐38 as compared to LoVo cells co‐implanted with control WI‐38 cells, or WI‐38 cells pretreated with Klotho (*n* = 6). Error bars were represented as mean ± SD. *P*‐values were analyzed with one‐way ANOVA. *P <* *0.05, **P <* *0.01, and ***P <* *0.001 compared with wild with D‐sen. ^#^ *P *<* *0.05, ^##^ *P *<* *0.01, and ^###^ *P *<* *0.001 compared with D‐sen with KL pre+D.

**Figure 3:**
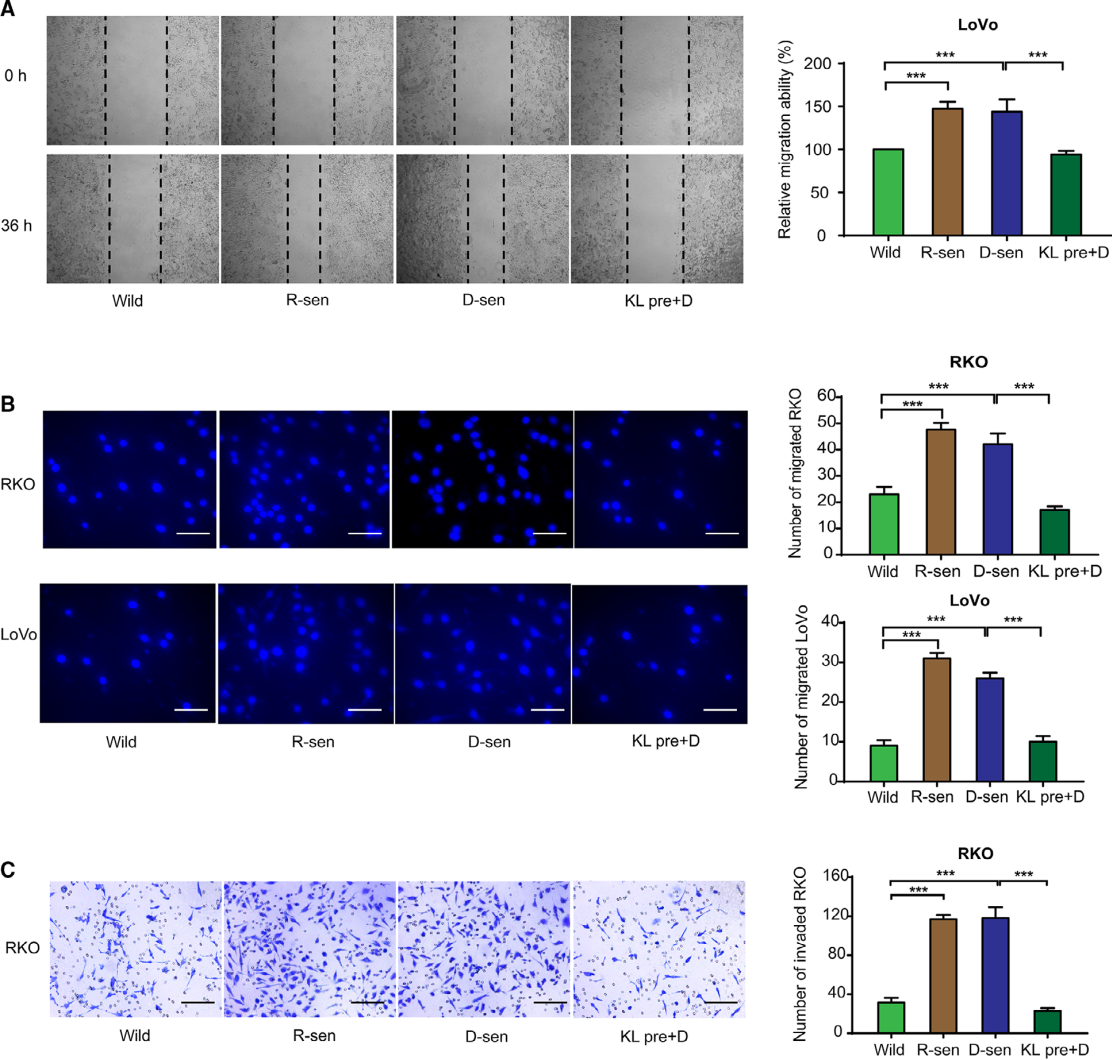
The effects of senescent fibroblasts and Klotho on CRC cell migration and invasion *in vitro*. Representative images of experimental wound healing (A), transwell migration (B), and Matrigel invasion (C) assays are shown. The results confirmed that replicative (R‐sen) or DOX‐induced (D‐sen) senescent WI‐38 could promote CRC cell migration and invasion, while this effect was blocked by exogenous administration with Klotho. Scale bars for B: 100 μm, 40× magnification. Scale bars for C: 200 μm, 20× magnification. Error bars were represented as mean ± SD (*n* = 3). *P*‐values were analyzed with one‐way ANOVA. ***P <* *0.001.

It has been previously suggested that a senescent cellular microenvironment can impact tumor progression (Campisi, 2013). To evaluate whether senescent stromal cells contribute to the progression of CRC, two methods leading to cell senescence were evaluated. Replicative‐ or DOX‐induced cellular senescence were used. WI‐38 and HUVEC were treated with doxorubicin to induce senescence (D‐sen). Long‐term growth was used for inducing replicative senescence and was used here as a positive control (R‐sen). Morphologic features of senescence including cell enlargement and flattening were microscopically detected. A commercial β‐gal stain for senescence was then evaluated in the DOX‐treated cells and R‐sen groups (Fig. 1A,B). A significant induction of the senescence markers p21 and p53 was detected at both mRNA and protein expression levels in the DOX‐treated and R‐sen groups (Fig. 1C,D). Conditioned medium (CM) was then collected from the senescent cells and used to treat CRC cells (RKO and LoVo), and the potential effect on growth characteristics was evaluated. The results show that CM from replicative or DOX‐induced senescent cells could enhance CRC cell proliferation, as demonstrated by the cell growth curves (Fig. 2A and Fig. S1A), and colony formation assays (Fig. 2B and Fig. S1B). To validate these studies *in vivo,* subcutaneous co‐implantation of tumor cells with senescent WI‐38 fibroblasts was performed. The results found enhanced tumor formation and growth in nude mice when senescent WI‐38 cells were used (Fig. 2C). In addition, using modified Boyden chamber assays we could show that CM from senescent stromal cells significantly enhanced the migration of CRC cell (RKO and LoVo) and enhanced the invasion CRCs (Fig. 3 and Fig. S2).

### 3.2 Klotho suppresses colon cancer progression by attenuating the DOX‐induced senescent phenotype

The studies described above have suggested that senescent stromal cells may help promote the malignant behavior of CRC cells. Klotho, a widely recognized anti‐aging protein, was previously proposed to act as a tumor suppressor in CRC (Arbel Rubinstein *et al*., 2018; Pan *et al*., 2011). Pretreatment of the senescent WI‐38 with Klotho partially reversed the enhanced proliferation and invasion seen after treating the CRC lines with senescent WI‐38 CM. The growth stimulatory effect induced *in vivo* by senescent fibroblasts in experimental CRC tumors in nude mice was also blocked by the exogenous administration of Klotho (Figs 2 and 3, Figs S1 and S2). Pretreatment with recombinant human Klotho protein was found to attenuate the DOX‐induced senescence of stromal cells. The level of SA‐β‐gal cells, and the mRNA and protein expression of p21 and p53, was significantly reduced following Klotho pretreatment of the DOX‐induced cells (Fig. 1). These results suggest that the tumor‐suppressing effects of Klotho may be mediated in part by attenuation of stromal cell senescence.

### 3.3 CCL2 is a SASP candidate in the senescent microenvironment

**Figure 4:**
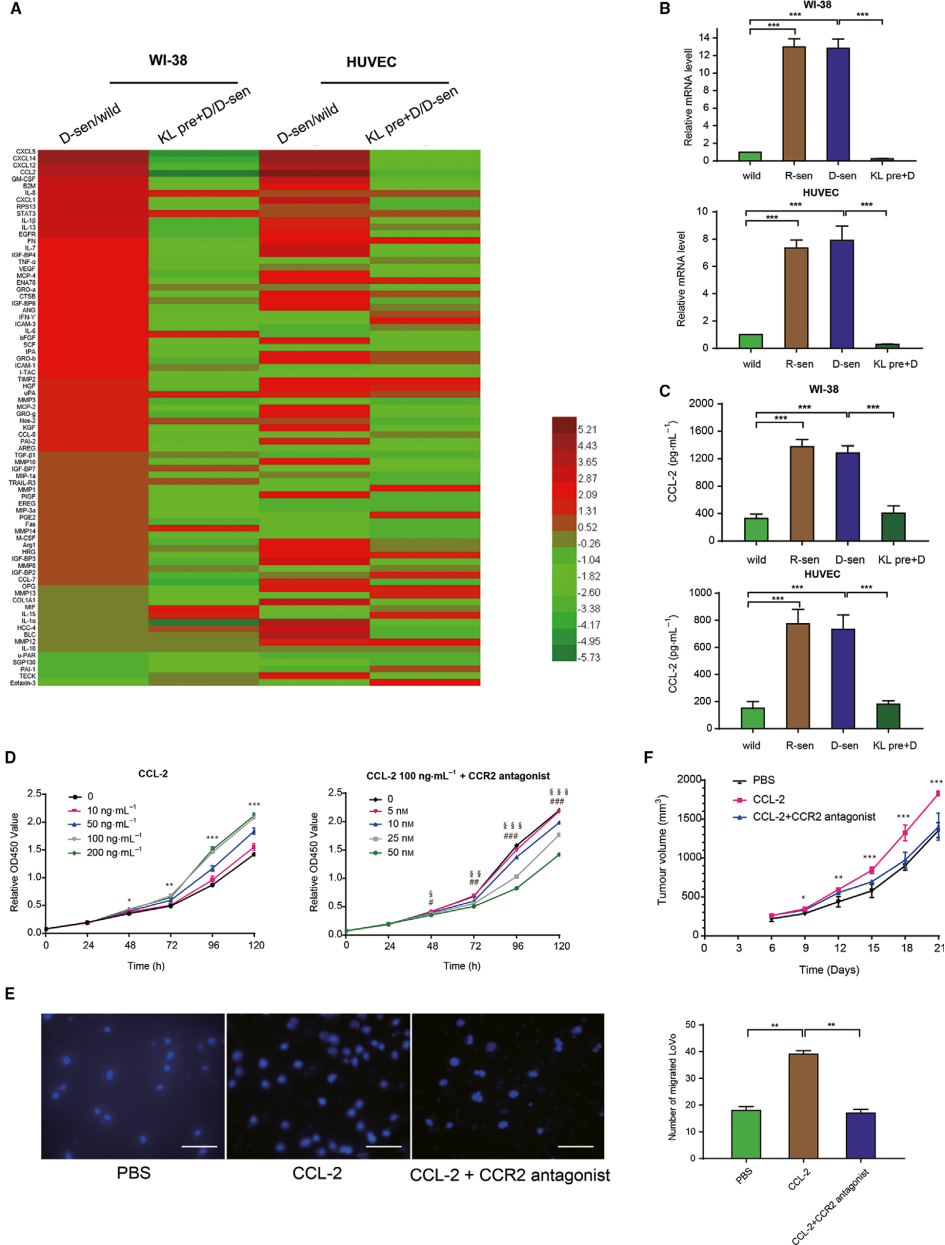
CCL2 is a SASP candidate in the senescent microenvironment. Screening candidate secreted factors up‐regulated in senescent stromal cells and down‐regulated by Klotho. (A) Real‐time PCR for common SASP genes selected candidates, which were significantly upregulated in senescent stromal cells and altered in cells pretreated with Klotho. The heatmap was generated by heatmap illustrator software using the relative values of D‐sen versus wild and KL pre+D versus D‐sen. Relative mRNA (B) and protein (C) levels of CCL2 in WI‐38 and HUVEC cells were evaluated by real‐time PCR and ELISA, respectively. GAPDH was used as an internal control. ***P <* *0.001. (D) Cell viability assay showed that CCL2 promoted CRC cells proliferation in a dose‐dependent manner, while CCR2 antagonist significantly blocked this effect. *P <* *0.05, **P <* *0.01, and ***P <* *0.001 compared with 100 ng·mL^−1^ CCL2 with control. ^§^ *P *<* *0.05, ^§§^ *P *<* *0.01, and ^§§§^ *P *<* *0.001 compared with 25 nm CCR2 antagonist with control. ^#^ *P *<* *0.05, ^##^ *P *<* *0.01, and ^###^ *P *<* *0.001 compared with 50 nm CCR2 antagonist with control. Similar results were observed in cell migration assay (E) and tumor growth in nude mice (F). *P <* *0.05, **P <* *0.01, and ***P <* *0.001 compared with CCL2 with PBS. Scale bars for E: 100 μm, 40× magnification. Error bars were represented as mean ± SD (*n* = 3). *P*‐values were analyzed with one‐way ANOVA.

**Figure 5:**
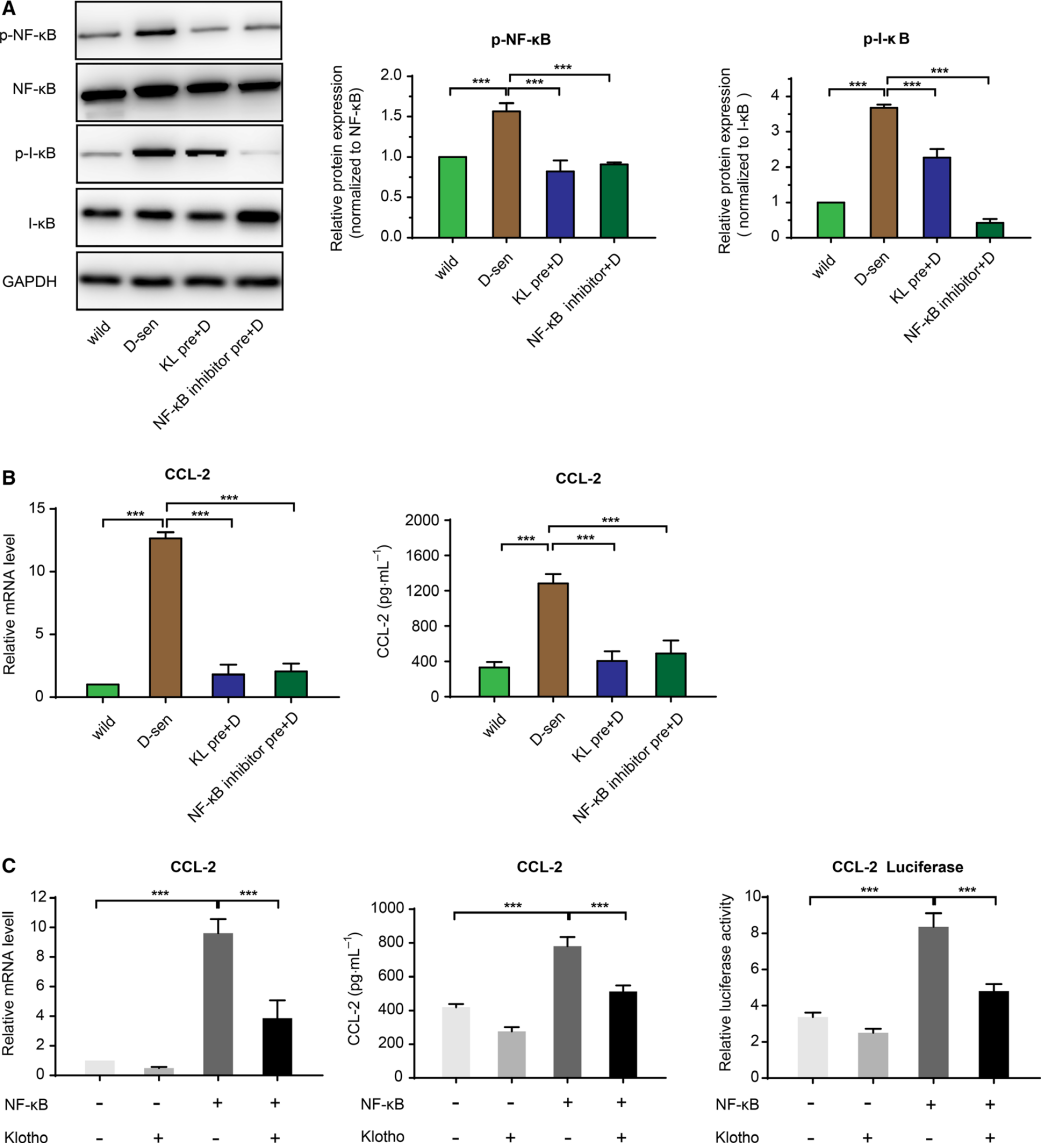
Klotho inhibits CCL2 secretion through modulation of the NF‐κB signaling pathway. (A) Western blot analysis was used to detect the phosphorylation of NF‐κB and I‐κB expression in wild‐type WI‐38 and WI‐38 cells pretreated with DOX, Klotho and NF‐κB inhibitor, and the relative protein expression was determined. (B) Relative mRNA and protein levels of CCL2 in WI‐38 cells with indicated treatment were evaluated by real‐time PCR and ELISA, respectively. (C) HEK293 cells were transfected with plasmids as indicated. Total RNA and supernatant were collected and subjected to analysis by real‐time PCR and a reporter assay for CCL2 activity, respectively. Error bars were represented as mean ± SD (*n* = 3). *P*‐values were analyzed with one‐way ANOVA. ***P <* *0.001.

The SASP present in the senescent stromal cells was then characterized to identify soluble factors that could potentially drive the tumorigenic effects seen in experimental CRC. The steady‐state mRNA expression of a panel of genes previously reported to be associated with SASP (Coppé *et al*., 2010, Table S2) was evaluated in the senescent and control cells using qPCR. The steady‐state levels of four candidate chemokines (CXCL12, CXCL14, IL‐8, and CCL2) were found to be strongly upregulated in the senescent stromal cells, and to also be reduced in mRNA expression in experimental stromal cells that were pretreated with Klotho (Fig. 4A–C and Table 1). The four chemokines identified are strongly linked to directed cell migration. Of particular interest was the chemokine CCL2, which was previously reported to be associated with the clinical progression of CRC (Chun *et al*., 2015). We observed that recombinant CCL2 could promote the proliferation and migration CRC cells *in vitro,* and enhance tumourigenesis *in vivo*. While administration of a CCR2 antagonist, a small molecule inhibitor of C‐C chemokine receptor 2 blocked these effects in the CRC cells tested (Fig. 4D–F). Notably, higher levels of CCL2 were measured in CM taken from senescent stromal cells (WI‐38 and HUVEC) as compared to nonsenescent cells, or cells pretreated with Klotho using specific ELISA (Fig. 5B). Adding recombinant CCL2 to the CM taken from nonsenescent WI‐38 cells enhanced the proliferation and migration of the RKO and LoVo cell lines (Fig. S3). Interestingly, treatment with recombinant CXCL12, another highly upregulated SASP‐chemokine from senescent stromal cells, did not influence cell viability or migration of the LoVo cell line (Fig. S4). Collectively, the results suggest that CCL2 may represent a candidate SASP protein in senescent stromal cells, and may play an important role in modulation of the malignant phenotype of CRC cells.

**Table 1:** The top 10 significantly changed SASP molecules in different cells. D‐sen/wild: The fold change of DOX‐induced senescent cell versus wild cell; KL pre+D/D‐sen: The fold change of Klotho‐pretreated cell versus DOX‐induced senescent cell.

WI‐38				HUVEC			
SASP	D‐sen/wild (Fold change)	SASP	KL pre+D/D‐sen (Fold change)	SASP	D‐sen/wild (Fold change)	SASP	KL pre+D/D‐sen (Fold change)
CXCL5	27.60	CXCL12	0.02	CXCL12	63.84	CCL7	0.10
CXCL14	19.09	IL‐1α	0.02	CXCL5	31.85	CXCL12	0.12
CXCL12	16.12	CXCL5	0.08	CXCL14	20.51	IL‐8	0.15
CCL2	12.32	CCL7	0.09	FN	14.81	CXCL14	0.17
GM‐CSF	10.16	CXCL14	0.14	IL‐1α	14.46	MCP‐2	0.17
B2M	9.63	MCP‐2	0.15	IL‐8	8.62	CCL2	0.20
IL‐8	9.39	IL‐1β	0.16	CCL2	7.90	IL‐6	0.21
CXCL1	9.20	CCL2	0.19	COL1A1	7.59	Nos‐2	0.23
RPS‐13	8.27	IL‐8	0.21	IGF‐BP3	7.45	IL‐1α	0.24
STAT3	7.96	KGF	0.23	HCC‐4	7.36	CCL8	0.24

### 3.4 Klotho regulates the secretion of CCL2 through modulation of the NF‐κB signaling pathway

We then sought to better characterize the interaction between Klotho and CCL2, as it relates to possible signaling pathways involved in this effect. DOX‐induced senescence was found to be associated with activated NF‐κB signaling as shown by increased levels of NF‐κB and I‐κB phosphorylation in the senescent cells (Fig. 5A). Klotho pretreatment suppressed this protein phosphorylation in a way that was similar to that seen with the NF‐κB inhibitor Bay 117085 (Fig. 5A). Additionally, the DOX‐senescent/induced CCL2 secretion could be blocked by a NF‐κB inhibitor or by Klotho pretreatment of the fibroblast cells (Fig. 5B). To investigate whether Klotho could directly regulate CCL2 secretion through modulation of the NF‐κB pathway, HEK293 cells were transfected with Klotho and various NF‐κB‐related expression plasmids, and the subsequent effect on CCL2 expression determined. Overexpression of NF‐κB components led to higher mRNA expression and secreted protein levels of CCL2, whereas overexpression of Klotho attenuated the NF‐κB‐associated CCL2 expression. NF‐κB‐driven luciferase reporter gene analysis showed that NF‐κB signaling, which is well associated with CCL2 transcription, could also be suppressed by ectopic expression of Klotho (Fig. 5C). These results strongly suggest that Klotho may regulate expression CCL2 via effects mediated through the NF‐κB signaling pathway.

### 3.5 Klotho and CCL2 were correlated with overall survival of CRC patients

**Figure 6:**
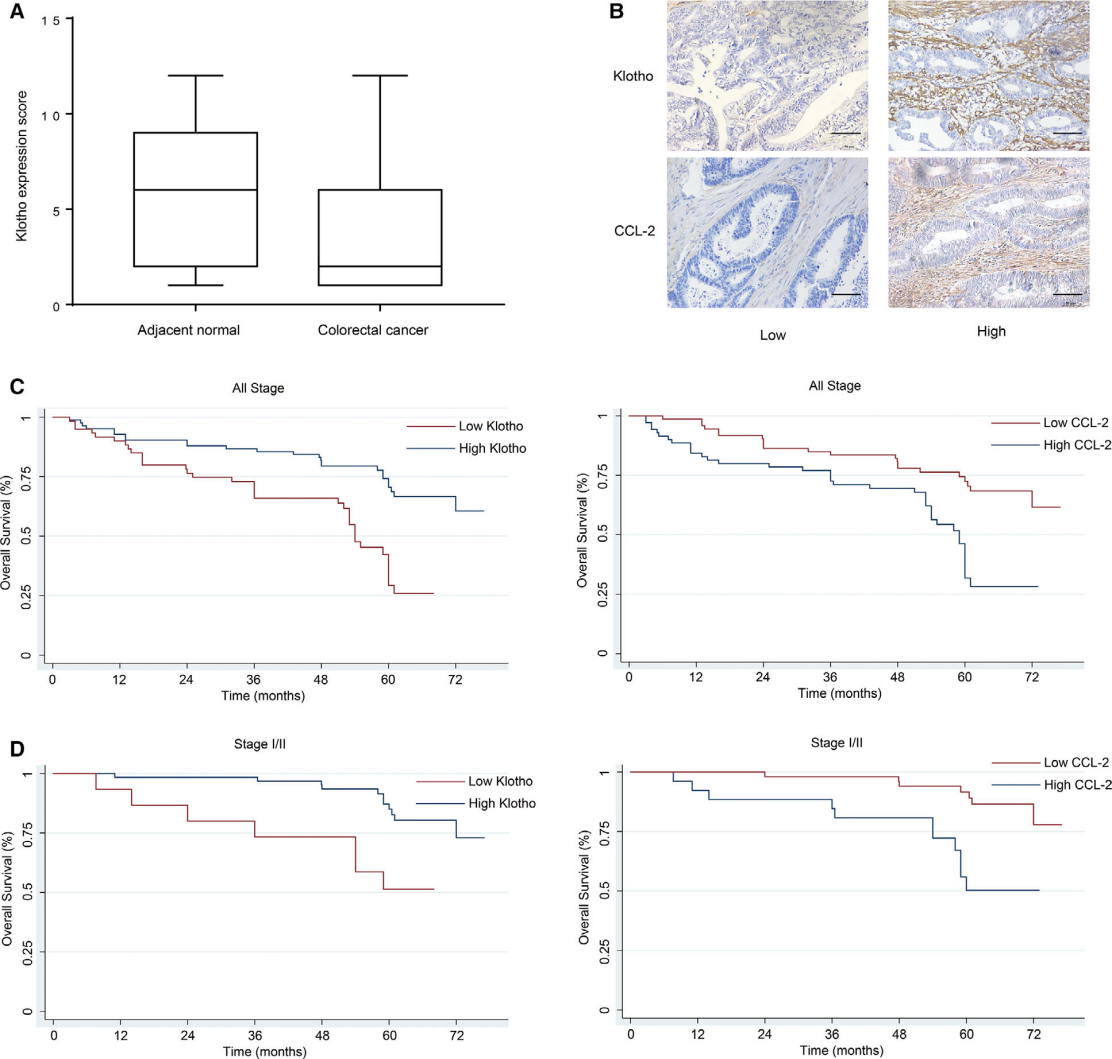
Klotho is down‐regulated in CRC and associated with poor prognosis. (A) The expression of Klotho in CRC tumor tissues and matched adjacent normal tissues was determined by IHC. The expression score showed that Klotho expression was significantly reduced in CRC tumor tissues. (B) Representative examples of staining images of cancer tissue samples with high or low expression of Klotho and CCL2 were shown. Scale bar: 200 μm, 20× magnification. (C) Kaplan–Meier survival analysis in patients from all stages indicated that CRC patients with a higher expression of Klotho, or lower expression of CCL2, had significantly longer survival (Klotho: *P *<* *0.001; CCL2: *P *=* *0.001). (D) Further stratification revealed that high expression of Klotho or low expression of CCL2 predicted even more favorable survival in patients with early TNM stage (I/II) (Klotho: *P* < 0.001; CCL2: *P* = 0.002).

To explore the potential clinical relevance of this biology, we then analyzed the expression of Klotho and CCL2 proteins in tissue samples from a cohort of CRC patients (Table S1). Immunohistochemical staining revealed that both Klotho and CCL2 are expressed primarily in the stroma of the tumor tissue. Klotho was found to be significantly reduced in CRC tissues as compared to adjacent normal tissues (*P *<* *0.01) (Fig. 6A). Representative images of the stained cancer tissue samples showing high or low expression of Klotho and CCL2 are shown in Fig. 6B. The expression of Klotho positively correlated with patient age, lymphatic metastasis, distant metastasis, and late TNM stage (*P *<* *0.05) (Table S1). After adjusting for potential confounding factors such as age, gender, differentiation, and TNM stage, Klotho expression was found to be an independent prognostic factor for overall survival (HR: 0.356, 95% CI: 0.08–0.581, *P *=* *0.03) as seen by multivariate Cox regression analysis (Table 2). In addition, Kaplan–Meier analysis showed a positive correlation between the expression of Klotho and CCL2, suggesting that CRC patients with higher expression Klotho or lower CCL2 have significantly longer survival. This difference was even more pronounced in patients of early stage I/II as compared to patients of all other stages (Fig. 6C,D).

**Table 2:** Predictors of survival identified by COX regression analysis.

Variables	Univariate COX regression			Multivariate COX regression		
	HR	95% CI	*P*‐value	HR	95% CI	*P*‐value
Age						
≤ 70	1			1		
> 70	2.505	0.662–4.419	0.029	2.845	0.443–18.250	0.040
Gender						
Female	1			1		
Male	1.767	0.819–3.813	0.147	0.841	0.156–4.539	0.840
Differentiation						
Well/Moderate	1			1		
Poor	3.919	1.269–12.107	0.018	2.180	0.60–7.921	0.237
Lymph node metastasis						
Negative	1					
Positive	4.724	0.839–8.851	0.026			
Distant metastasis						
Yes	1					
No	0.067	0.031–0.148	< 0.001			
TNM stage						
I–II	1			1		
III–IV	4.023	1.768–9.155	0.001	2.541	1.423–15.251	0.03
Klotho expression						
Low	1			1		
High	0.271	0.123–0.598	0.001	0.356	0.08–0.581	0.03

## 4 Discussion

Permissive microenvironments help support and promote tumor growth and aggressiveness. Cellular senescence, which occurs in response to excessive extracellular or intracellular stress, is thought to act as a protective mechanism against tumorigenesis. Recent reports have suggested that senescence is not irreversible, and may also promote tumor progression in certain settings. Several studies have reported the ability of senescent human stromal cells to promote the growth and tumorigenesis of premalignant and malignant lung cancer cells (Bartling *et al*., 2006; Lugo *et al*., 2016; Papadopoulou and Kletsas, 2011). Ruhland *et al*. suggested a mechanism whereby senescent stromal cells drive the local recruitment of immune suppressive myeloid cells that can promote tumor progression. These stromal cells can thus be seen to help establish an immune privileged microenvironment that allows tumor cells to proliferate (Ruhland *et al*., 2016). To better characterize the apparent paradoxical role that senescence plays in tumorigenesis, our study sought to study the impact of senescent stromal cells on the progression of CRC. We found that tumor cells cultured with conditioned medium from DOX‐induced or replicative senescent stromal cells (WI‐38 and HUVEC) showed higher proliferation, migration, and invasion *in vitro* and *in vivo*. Subcutaneous co‐implantation of CRC cells with senescent WI‐38 fibroblasts increased LoVo colon tumor formation and growth in nude mice. These observations strongly suggest that senescent stromal cells may promote the tumorigenesis and invasion of colon cancer cells. Importantly, we found that the pretreatment of tumor cells with conditional medium (CM) from senescent cells resulted in a long‐term effect on experimental tumor growth *in vivo*. Although the molecular basis of this complex interaction between the tumor and tumor microenvironment is at present unclear, this long‐acting effect may result from the modulation of key signaling pathways in the tumors that are altered by factors in the CM.

Although showing arrested growth, senescent cells are still metabolically active and have undergone changes in gene expression and protein secretion reflected by the expression of SASP (Coppé *et al*., 2010). The altered expression of diverse soluble and insoluble SASP factors is thought to modulate various signaling pathways that can impact tumor development and progression. Potential mechanisms linked to this process have been described in the literature where SASP factors were shown to support tumor cell invasion and metastasis in part by disrupting and remodeling the tissue structure (Coppé *et al*., 2008; Rodier and Campisi, 2011). SASP generated from senescent cells can also influence tumor vascularization, a key process associated with tumor progression (Davalos *et al*., 2010; Kelly *et al*., 2007). Finally, SASP was suggested to enhance tumor growth by fostering a microenvironment that is more immunosuppressive (Toso *et al*., 2014).

To help identify potential SASP candidate factors within the senescence microenvironment, we performed a qPCR screen using a panel of genes previously reported to be associated with SASP, and identified a series of SASP‐associated genes significantly upregulated in senescent stromal cells in our experimental setting, which included the chemokine CCL2. We could show that the increased secretion level of CCL2 from senescent stromal cells, or the exogenous administration of recombinant CCL2, could enhance the proliferation and invasion of RKO and LoVo cells *in vitro*. The enhanced effect was blocked by a CCR2‐specific receptor antagonist *in vitro*. In addition, the tumor‐promoting effect of senescent stromal cells seen in experimental tumor models in mice could also be reduced by administration of the CCR2 antagonist *in vivo*. These results strongly suggest that CCL2 may represent a key mediator secreted by senescent stromal cells, linked to the senescence microenvironment that can drive progression of CRC. In support of this hypothesis, steady‐state mRNA CCL2 levels were previously shown to be significantly elevated in colon cancer tissues as compared to normal colon (Ohlsson *et al*., 2016). The potential impact of this biology on various tumor‐associated phenomenon, such as tumor heterogeneity, is unknown requiring more detailed investigation.

We could show that Klotho can help attenuate DOX‐induced senescence in stromal cells and through this, suppress progression of experimental CRC. Klotho, a classical anti‐aging protein, plays critical role in the aging process and in the development of age‐related diseases including cancers (Zhou and Wang, 2015). Our previous work has shown that Klotho is silenced, or significantly down‐regulated in colon cancer cells by DNA hypermethylation in its promoter region. Klotho can inhibit colon cancer cell growth and their apoptosis (Pan *et al*., 2011). There are at least two forms of Klotho protein, a transmembrane form which generally expressed in the kidney and functions as a co‐receptor for FGF23, and a secreted form found in the peripheral circulation (Kuro‐o, 2009). In our study, we found that the pro‐tumorigenic effects induced by senescent stromal cells could be attenuated by exogenous administration with the soluble form of Klotho. Klotho protein expression was found to be significantly reduced in CRC patient samples as compared to adjacent normal tissues. The level of protein expression of Klotho correlated with distant metastasis and TNM stage and was found to act as an independent prognostic factor for survival outcome of CRC patients. These findings provide important new insight into the potential role of Klotho where it may act as a tumor suppressor within the senescent microenvironment of CRC.

Several signaling pathways have been found to play crucial roles in the process of senescence and the modulation of SASP. These include the MAP kinase and oxidative stress pathways (Fridman and Tainsky, 2008). We could confirm an increase in NF‐κB phosphorylation during fibroblast cells senescence and could verify that NF‐κB eventually controls relevant transcriptional targets. The DOX‐senescent induced CCL2 secretion seen in fibroblasts cells was effectively blocked by treatment with aNF‐κB inhibitor. Reduced NF‐κB signaling was reported to suppress SASP and improve chemotherapy efficiency in a mouse model of lymphoma (Chien *et al*., 2011). The authors found that NF‐κB was activated during senescence, which could in turn influence the DNA damage response, and sequentially trigger expression of SASP. The NF‐κB and Klotho signaling pathways have also been discussed in a recent study, showing that inflammation‐associated factors inhibit Klotho gene expression in CRC cells through activation of NF‐κB signaling (Xie *et al*., 2019). Klotho has also been described to prevent the degradation of IκB, an important regulator of NF‐κB, and thus block NF‐κB p65 nuclear translocation (Guo *et al*., 2018). The exact molecular mechanisms underlying Klotho regulation of NF‐κB signaling remain to be identified.

## 5 Conclusion

In conclusion, the results presented here, in concert with the studies highlighted above, strongly suggest that the senescent microenvironment has an impact on tumor growth, and potentially impacts its response to chemotherapy. We show that senescent stromal cells can promote the progression of experimental CRC. This effect can be suppressed by treatment with soluble Klotho, which was subsequently shown to inhibit secretion of the chemokine CCL2 in the senescent cells. CCL2, acting through its receptor CCR2, was found drive important aspects of senescence. The identification of SASP, including CCL2, may provide potential therapeutic targets in CRC as well as enhance our understanding of this important phenomenon in the context of tumor progression.

## Conflict of interest

The authors declare no conflict of interest.

## Author contributions

LW and SC made substantial contributions to the conception and design of this study. YL, LW, JP, JB, ZL, MX, and TS performed experiments, statistical analyses, and made the figures. YL, SL, FC, QG, LC, SY, and YZ analyzed and interpreted the data. JP, YL, and XP wrote the manuscript and performed language editing for the manuscript. LW helped write and revised the manuscript, providing intellectual feedback needed for the manuscript. All authors reviewed and agreed to the information in this manuscript.

## Supporting information


**Fig. S1**. The effects of senescent fibroblasts and Klotho on CRC cell growth. (**A**) Cell viability assay of cell lines RKO and LoVo showed that CM from replicative (R‐sen) or DOX‐induced (D‐sen) senescent HUVEC promoted cancer cell proliferation, while pretreatment with Klotho (KL pre+D) inhibited CRC cell growth. *p <* *0.05, **p <* *0.01 and *** *p *<* *0.001 compared with wild with D‐sen. ^#^ *p *<* *0.05, ^##^ *p *<* *0.01 and ^###^ *p *<* *0.001 compared with D‐sen with KL pre+D. Similar results were obtained from colony formation in both cell lines (**B**). *** indicates of *p *<* *0.001. Error bars were represented as mean ± SD (n = 3). *p*‐values were analyzed with one‐way ANOVA.

**Fig. S2.** The effects of senescent fibroblasts and Klotho on CRC cell migration and invasion. Representative images of wound healing (**A**), transwell migration (**B**) and Matrigel invasion (**C**) assays were shown. The calculation confirmed that replicative (R‐sen) or DOX‐induced (D‐sen) senescent HUVEC promoted CRC cell migration and invasion, while this effect was blocked by the exogenous administration with Klotho. Scale bars for B: 100 μm, 40× magnification. Scale bars for C: 200 μm, 20× magnification. Error bars were represented as mean ± SD (n = 3). *p*‐values were analyzed with one‐way ANOVA. ***indicates of *p *<* *0.001.

**Fig. S3.** The effects of recombinant CCL2 added to conditioned media from non‐senescent WI‐38 on CRC cell proliferation and migration. (**A**,** B**) Cell viability assay of cell lines RKO and LoVo showed that administrating recombinant CCL2 to CM of non‐senescent WI‐38 promoted cancer cell proliferation. Similar results were obtained from migration assay in both cell lines (**C**,** D**). Scale bars: 100 μm, 40× magnification. Error bars were represented as mean ± SD (n = 3). *p*‐values were analyzed with paired independent Student t test. * indicates of *p *<* *0.05; ** indicates of *p *<* *0.01 and *** indicates of *p *<* *0.001.

**Fig. S4.** The effect of CXCL12 on CRC cell proliferation and migration. (**A**) Real‐time PCR showed that CXCR4, the receptor of CXCL12, is highly expressed in LoVo cells as compared to the other CRC cell lines. (**B**) Cell viability assay showed that addition of recombinant CXCL12 at 0, 100, and 1000 ng/ml final concentration did not influence the LoVo cancer cell proliferation. Cell migration was also evaluated in LoVo cells (**C**). Scale bars: 100 μm, 40× magnification. Error bars were represented as mean ± SD (n = 3). *p*‐values were analyzed with one‐way ANOVA.

**Table S1.** The association of Klotho expression with clinicopathological features inpatients with CRC.
**Table S2.** The senescence‐associated secretory phenotype (SASP) associated genes.

## References

[mol212577-bib-0001] Agrawal K , Das V , Táborská N , Gurský J , Džubák P and Hajdúch M (2018) Differential regulation of methylation‐regulating enzymes by senescent stromal cells drives colorectal cancer cell response to DNA‐demethylating Epi‐drugs. Stem Cells Int 12, 6013728. 10.1155/2018/6013728 PMC6109465 30158986

[mol212577-bib-0002] Aird KM and Zhang R (2015) Nucleotide metabolism, oncogene‐induced senescence and cancer. Cancer Lett 356, 204–210. 24486217 10.1016/j.canlet.2014.01.017 PMC4115046

[mol212577-bib-0003] Arbel Rubinstein T , Shahmoon S , Zigmond E , Etan T , Merenbakh‐Lamin K , Pasmanik‐Chor M , Har‐Zahav G , Barshack I , Vainer GW , Skalka N *et al* (2018) Klotho suppresses colorectal cancer through modulation of the unfolded protein response. Oncogene 38, 794–807. 30232408 10.1038/s41388-018-0489-4

[mol212577-bib-0004] Bartling B , Demling N , Silber RE and Simm A (2006) Proliferative stimulus of lung fibroblasts on lung cancer cells is impaired by the receptor for advanced glycation end‐products. Am J Respir Cell Mol Biol 34, 83–91. 16166741 10.1165/rcmb.2005-0194OC

[mol212577-bib-0005] Bhowmick NA , Neilson EG and Moses HL (2004) Stromal fibroblasts in cancer initiation and progression. Nature 432, 332–337. 15549095 10.1038/nature03096 PMC3050735

[mol212577-bib-0006] Campisi J (2013) Aging, cellular senescence, and cancer. Annu Rev Physiol 75, 685–705. 23140366 10.1146/annurev-physiol-030212-183653 PMC4166529

[mol212577-bib-0007] Chien Y , Scuoppo C , Wang X , Fang X , Balgley B , Bolden JE , Premsrirut P , Luo W , Chicas A , Lee CS *et al* (2011) Control of the senescence‐associated secretory phenotype by NF‐κB promotes senescence and enhances chemosensitivity. Genes Dev 25, 2125. 21979375 10.1101/gad.17276711 PMC3205583

[mol212577-bib-0008] Chun E , Lavoie S , Michaud M , Gallini CA , Kim J , Soucy G , Odze R , Glickman JN and Garrett WS (2015) CCL2 promotes colorectal carcinogenesis by enhancing polymorphonuclear myeloid‐derived suppressor cell population and function. Cell Rep 12, 244–257. 26146082 10.1016/j.celrep.2015.06.024 PMC4620029

[mol212577-bib-0009] Collado M and Serrano M (2010) Senescence in tumours: evidence from mice and humans. Nat Rev Cancer 10, 51–57. 20029423 10.1038/nrc2772 PMC3672965

[mol212577-bib-0010] Coppé JP , Desprez PY , Krtolica A and Campisi J (2010) The senescence‐associated secretory phenotype: the dark side of tumor suppression. Annu Rev Pathol 5, 99–118. 20078217 10.1146/annurev-pathol-121808-102144 PMC4166495

[mol212577-bib-0011] Coppé JP , Patil CK , Rodier F , Sun Y , Muñoz DP , Goldstein J , Nelson PS , Desprez PY and Campisi J (2008) Senescence‐associated secretory phenotypes reveal cell‐nonautonomous functions of oncogenic RAS and the p53 tumor suppressor. PLoS Biol 6, 2853–2868. 19053174 10.1371/journal.pbio.0060301 PMC2592359

[mol212577-bib-0012] Courtois‐Cox S , Jones SL and Cichowski K (2008) Many roads lead to oncogene‐induced senescence. Oncogene 27, 2801–2809. 18193093 10.1038/sj.onc.1210950

[mol212577-bib-0013] Davalos AR , Coppe JP , Campisi J and Desprez PY (2010) Senescent cells as a source of inflammatory factors for tumor progression. Cancer Metastasis Rev 29, 273–283. 20390322 10.1007/s10555-010-9220-9 PMC2865636

[mol212577-bib-0014] Debacq‐Chainiaux F , Erusalimsky JD , Campisi J and Toussaint O (2009) Protocols to detect senescence‐associated beta‐galactosidase (SA‐beta gal) activity, a biomarker of senescent cells in culture and in vivo. Nat Protoc 4, 1798–1806. 20010931 10.1038/nprot.2009.191

[mol212577-bib-0015] Fridman AL and Tainsky MA (2008) Critical pathways in cellular senescence and immortalization revealed by gene expression profiling. Oncogene 27, 5975–5987. 18711403 10.1038/onc.2008.213 PMC3843241

[mol212577-bib-0016] Gordon RR and Nelson PS (2012) Cellular senescence and cancer chemotherapy resistance. Drug Resist Updat 15, 123–131. 22365330 10.1016/j.drup.2012.01.002 PMC3348393

[mol212577-bib-0017] Guo Y , Zhuang X , Huang Z , Zou J , Yang D , Hu X , Du Z , Wang L and Liao X (2018) Klotho protects the heart from hyperglycemia‐induced injury by inactivating ROS and NF‐κB‐mediated inflammation both in vitro and in vivo. Biochim Biophys Acta Mol Basis Dis 1864, 238–251. 28982613 10.1016/j.bbadis.2017.09.029

[mol212577-bib-0018] Ibi T , Usuda J , Inoue T , Sato A and Takegahara K (2017) Klotho expression is correlated to molecules associated with epithelial‐mesenchymal transition in lung squamous cell carcinoma. Oncol Lett 14, 5526–5532. 29142604 10.3892/ol.2017.6862 PMC5666650

[mol212577-bib-0019] Kelly J , Ali Khan A , Yin J , Ferguson TA and Apte RS (2007) Senescence regulates macrophage activation and angiogenic fate at sites of tissue injury in mice. J Clin Invest 117, 3421–3426. 17975672 10.1172/JCI32430 PMC2045608

[mol212577-bib-0020] Kuro‐o M (2009) Klotho and aging. Biochim Biophys Acta 1790, 1049–1058. 19230844 10.1016/j.bbagen.2009.02.005 PMC2743784

[mol212577-bib-0021] Kuro‐o M , Matsumura Y , Aizawa H , Kawaguchi H , Suga T , Utsugi T , Ohyama Y , Kurabayashi M , Kaname T , Kume E *et al* (1997) Mutation of the mouse klotho gene leads to a syndrome resembling ageing. Nature 390, 45–51. 9363890 10.1038/36285

[mol212577-bib-0022] Kurosu H , Yamamoto M , Clark JD , Pastor JV , Nandi A , Gurnani P , McGuinness OP , Chikuda H , Yamaguchi M , Kawaguchi H *et al* (2005) Suppression of aging in mice by the hormone Klotho. Science 309, 1829–1833. 16123266 10.1126/science.1112766 PMC2536606

[mol212577-bib-0023] Lasry A and Ben‐Neriah Y (2015) Senescence‐associated inflammatory responses: aging and cancer perspectives. Trends Immunol 36, 217–228. 25801910 10.1016/j.it.2015.02.009

[mol212577-bib-0024] Lee JS , Yoo JE , Kim H , Rhee H , Koh MJ , Nahm JH , Choi JS , Lee KH and Park YN (2017) Tumor stroma with senescence‐associated secretory phenotype in steatohepatitic hepatocellular carcinoma. PLoS One 12, e0171922. 28273155 10.1371/journal.pone.0171922 PMC5342190

[mol212577-bib-0025] Ligumsky H , Rubinek T , Merenbakh‐Lamin K , Yeheskel A , Sertchook R , Shahmoon S , Aviel‐Ronen S and Wolf I (2015) Tumor suppressor activity of Klotho in breast cancer is revealed by structure‐function analysis. Mol Cancer Res 13, 1398–1407. 26113466 10.1158/1541-7786.MCR-15-0141

[mol212577-bib-0026] Lojkin I , Rubinek T , Orsulic S , Schwarzmann O , Karlan BY , Bose S and Wolf I (2015) Reduced expression and growth inhibitory activity of the aging suppressor klotho in epithelial ovariancancer. Cancer Lett 362, 149–157. 25827069 10.1016/j.canlet.2015.03.035

[mol212577-bib-0027] Lugo R , Gabasa M , Andriani F , Puig M , Facchinetti F , Ramírez J , Gómez‐Caro A , Pastorino U , Fuster G , Almendros I *et al* (2016) Heterotypic paracrine signaling drives fibroblast senescence and tumor progression of large cell carcinoma of the lung. Oncotarget 7, 82324–82337. 27384989 10.18632/oncotarget.10327 PMC5347694

[mol212577-bib-0028] Ohlsson L , Hammarström ML , Lindmark G , Hammarström S and Sitohy B (2016) Ectopic expression of the chemokine CXCL17 in colon cancer cells. Br J Cancer 114, 697–703. 26889977 10.1038/bjc.2016.4 PMC4800305

[mol212577-bib-0029] Pan J , Zhong J , Gan LH , Chen SJ , Jin HC , Wang X and Wang LJ (2011) Klotho, an anti‐senescence related gene, is frequently inactivated through promoter hypermethylation in colorectal cancer. Tumour Biol 32, 729–735. 21523445 10.1007/s13277-011-0174-5

[mol212577-bib-0030] Papadopoulou A and Kletsas D (2011) Human lung fibroblasts prematurely senescent after exposure to ionizing radiation enhance the growth of malignant lung epithelial cells in vitro and in vivo. Int J Oncol 39, 989–999. 21814715 10.3892/ijo.2011.1132

[mol212577-bib-0039] Qian Y , Wong CC , Xu J , Chen H , Zhang Y , Kang W , Wang H , Zhang L , Li W , Chu ESH *et al* (2017) Sodium channel subunit scnn1b suppresses gastric cancer growth and metastasis via grp78 degradation. Cancer Res 77, 1968–1982. 28202509 10.1158/0008-5472.CAN-16-1595

[mol212577-bib-0031] Rodier F and Campisi J (2011) Four faces of cellular senescence. J Cell Biol 192, 547–556. 21321098 10.1083/jcb.201009094 PMC3044123

[mol212577-bib-0032] Ruhland MK , Loza AJ , Capietto AH , Luo X , Knolhoff BL , Flanagan KC , Belt BA , Alspach E , Leahy K , Luo J *et al* (2016) Stromal senescence establishes an immunosuppressive microenvironment that drives tumorigenesis. Nat Commun 7, 11762. 27272654 10.1038/ncomms11762 PMC4899869

[mol212577-bib-0033] Taddei ML , Cavallini L , Comito G , Giannoni E , Folini M , Marini A , Gandellini P , Morandi A , Pintus G , Raspollini MR *et al* (2014) Senescent stroma promotes prostate cancer progression: the role of miR‐210. Mol Oncol 8, 1729–1746. 25091736 10.1016/j.molonc.2014.07.009 PMC5528604

[mol212577-bib-0034] Toso A , Revandkar A , Di Mitri D , Guccini I , Proietti M , Sarti M , Pinton S , Zhang J , Kalathur M , Civenni G *et al* (2014) Enhancing chemotherapy efficacy in Pten‐deficient prostate tumors by activating the senescence‐associated antitumor immunity. Cell Rep 9, 75–89. 25263564 10.1016/j.celrep.2014.08.044

[mol212577-bib-0035] Wang T , Notta F , Navab R , Joseph J , Ibrahimov E , Xu J , Zhu CQ , Borgida A , Gallinger S and Tsao MS (2017) Senescent carcinoma‐associated fibroblasts upregulate IL8 to enhance prometastatic phenotypes. Mol Cancer Res 15, 3–14. 27678171 10.1158/1541-7786.MCR-16-0192

[mol212577-bib-0036] Wang L , Wang X , Wang X , Jie P , Lu H , Zhang S , Lin X , Lam EK , Cui Y , Yu J *et al* (2011) Klotho is silenced through promoter hypermethylation in gastric cancer. Am J Cancer Res 1, 111–119. 21969138 PMC3180103

[mol212577-bib-0037] Xie B , Nie S , Hu G , Xiong L , Hu F , Li M , Peng T , Nie J and He Y (2019) The involvement of NF‐κB/Klotho signaling in colorectal cancer cell survival and invasion. Pathol Oncol Res. 10.1007/s12253-018-0493-6. [Epub ahead of print]. 30612312

[mol212577-bib-0038] Zhou X and Wang X (2015) Klotho: a novel biomarker for cancer. J Cancer Res Clin Oncol 141, 961–969. 25086986 10.1007/s00432-014-1788-y PMC11823687

