# Supplementary figures and images for: Klotho‐mediated targeting of CCL2 suppresses the induction of colorectal cancer progression by stromal cell senescent microenvironments
Source: Mol Oncol. 2019 Oct 6;13(11):2460–75. doi: 10.1002/1878-0261.12577 (PMC6822285; doi:10.1002/1878-0261.12577)

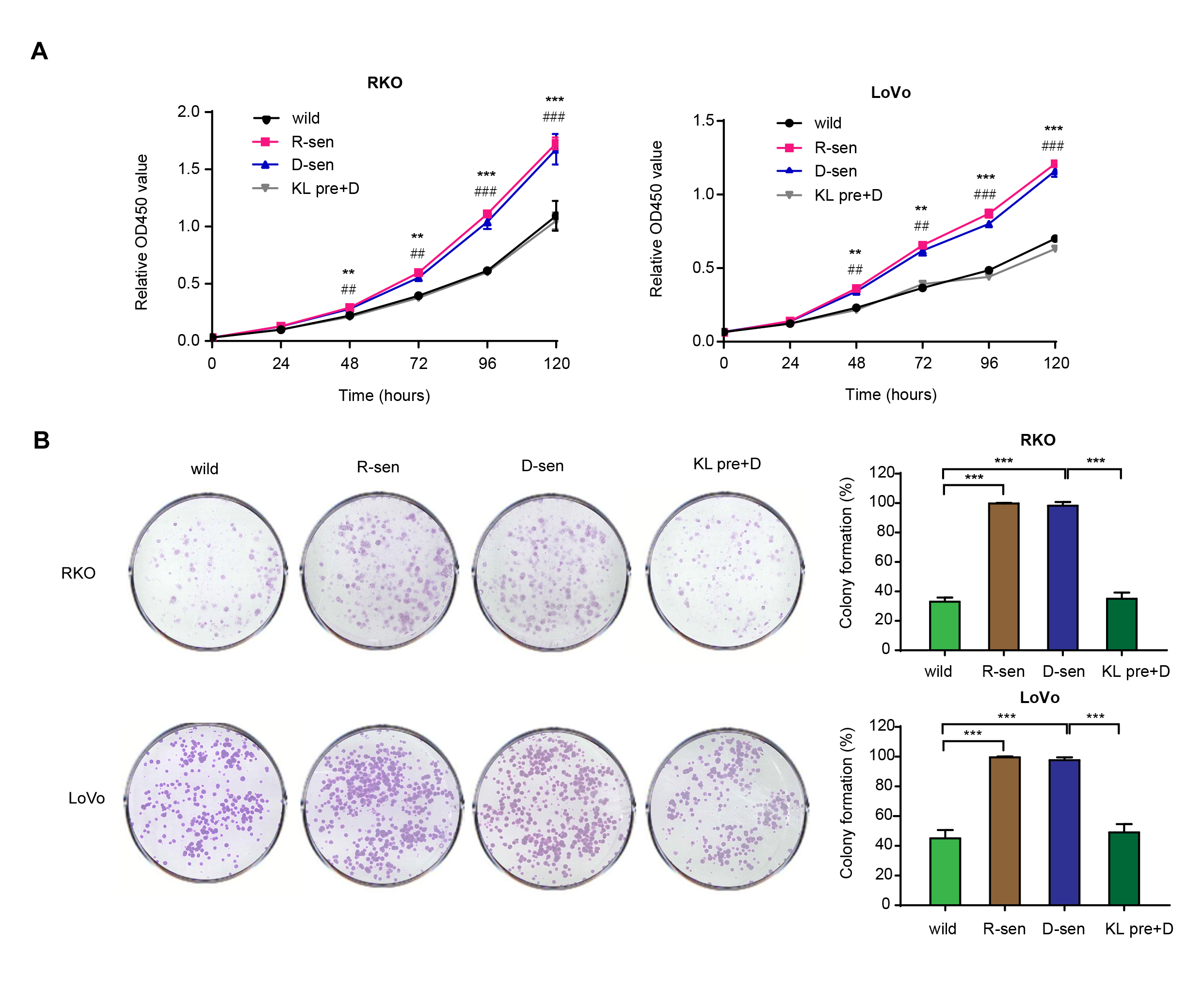

Supplement: Supplementary file 1 — Fig. S1. The effects of senescent fibroblasts and Klotho on CRC cell growth. (A) Cell viability assay of cell lines RKO and LoVo showed that CM from replicative (R‐sen) or DOX‐induced (D‐sen) senescent HUVEC promoted cancer cell proliferation, while pretreatment with Klotho (KL pre+D) inhibited CRC cell growth. *p < 0.05, **p < 0.01 and *** p < 0.001 compared with wild with D‐sen. # p < 0.05, ## p < 0.01 and ### p < 0.001 compared with D‐sen with KL pre+D. Similar results were obtained from colony formation in both cell lines (B). *** indicates of p < 0.001. Error bars were represented as mean ± SD (n = 3). p‐values were analyzed with one‐way ANOVA. [file MOL2-13-2460-s001.tif]

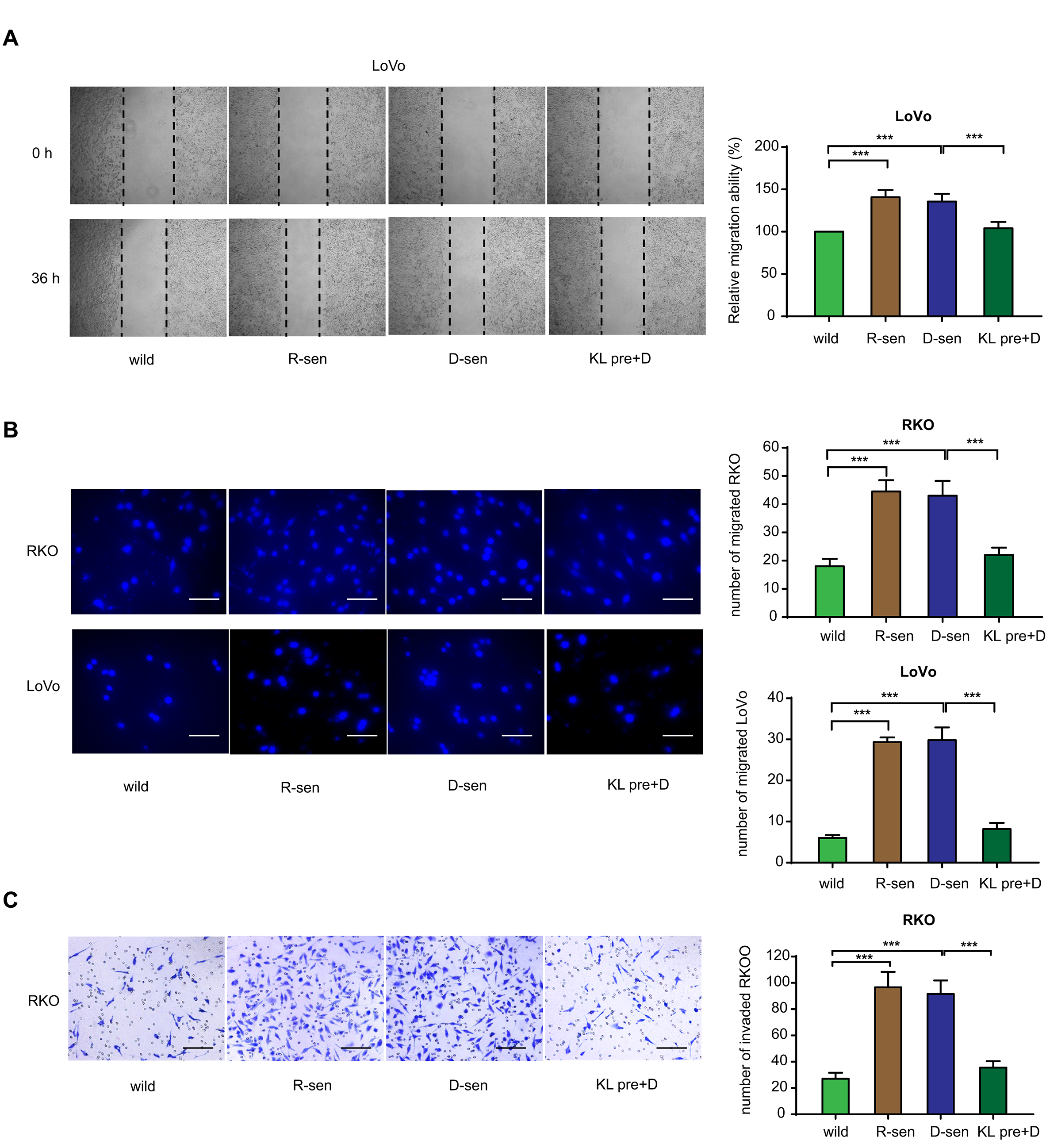

Supplement: Supplementary file 2 — Fig. S2. The effects of senescent fibroblasts and Klotho on CRC cell migration and invasion. Representative images of wound healing (A), transwell migration (B) and Matrigel invasion (C) assays were shown. The calculation confirmed that replicative (R‐sen) or DOX‐induced (D‐sen) senescent HUVEC promoted CRC cell migration and invasion, while this effect was blocked by the exogenous administration with Klotho. Scale bars for B: 100 μm, 40× magnification. Scale bars for C: 200 μm, 20× magnification. Error bars were represented as mean ± SD (n = 3). p‐values were analyzed with one‐way ANOVA. ***indicates of p < 0.001. [file MOL2-13-2460-s002.tif]

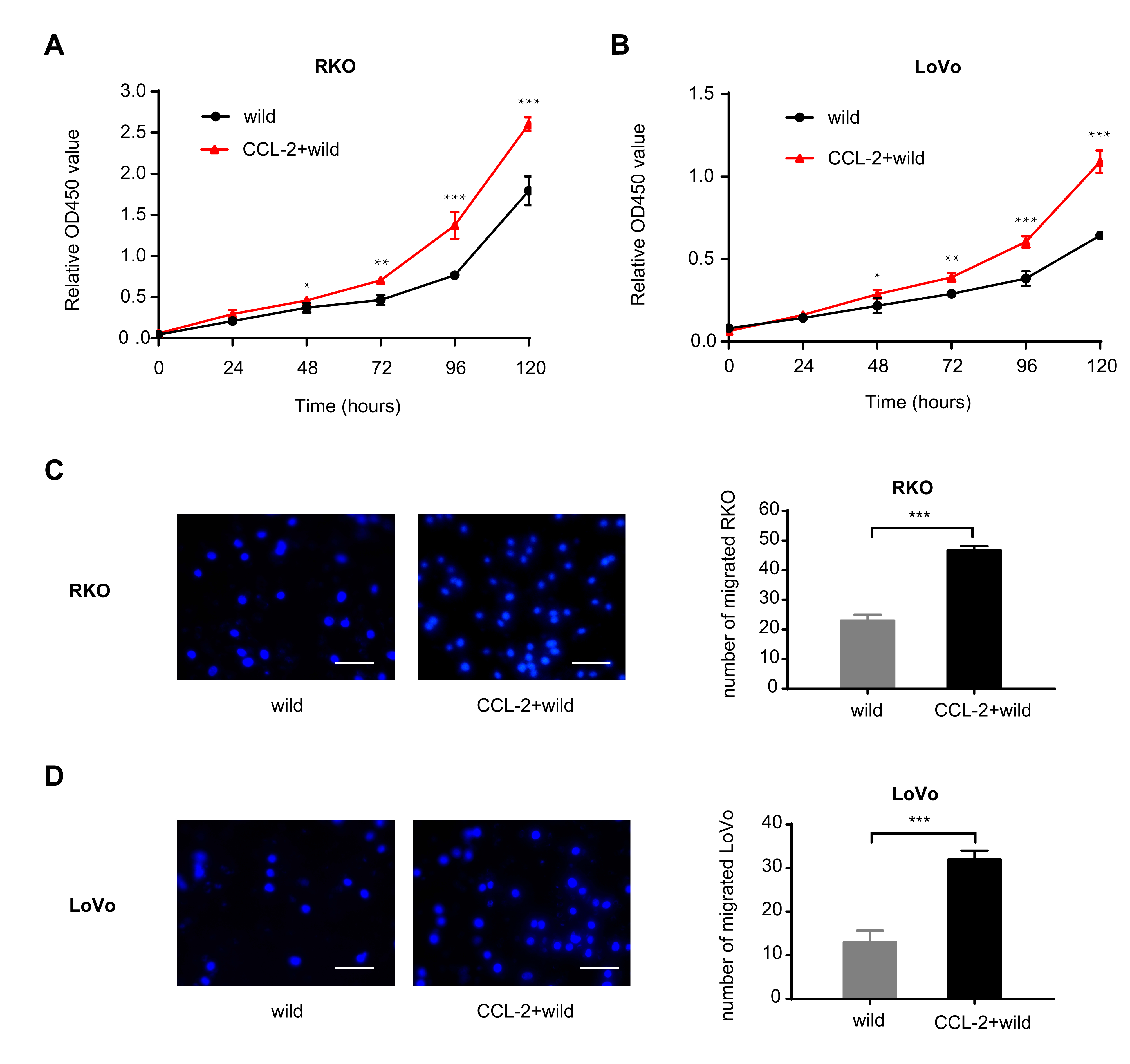

Supplement: Supplementary file 3 — Fig. S3. The effects of recombinant CCL2 added to conditioned media from non‐senescent WI‐38 on CRC cell proliferation and migration. (A, B) Cell viability assay of cell lines RKO and LoVo showed that administrating recombinant CCL2 to CM of non‐senescent WI‐38 promoted cancer cell proliferation. Similar results were obtained from migration assay in both cell lines (C, D). Scale bars: 100 μm, 40× magnification. Error bars were represented as mean ± SD (n = 3). p‐values were analyzed with paired independent Student t test. * indicates of p < 0.05; ** indicates of p < 0.01 and *** indicates of p < 0.001. [file MOL2-13-2460-s003.tif]

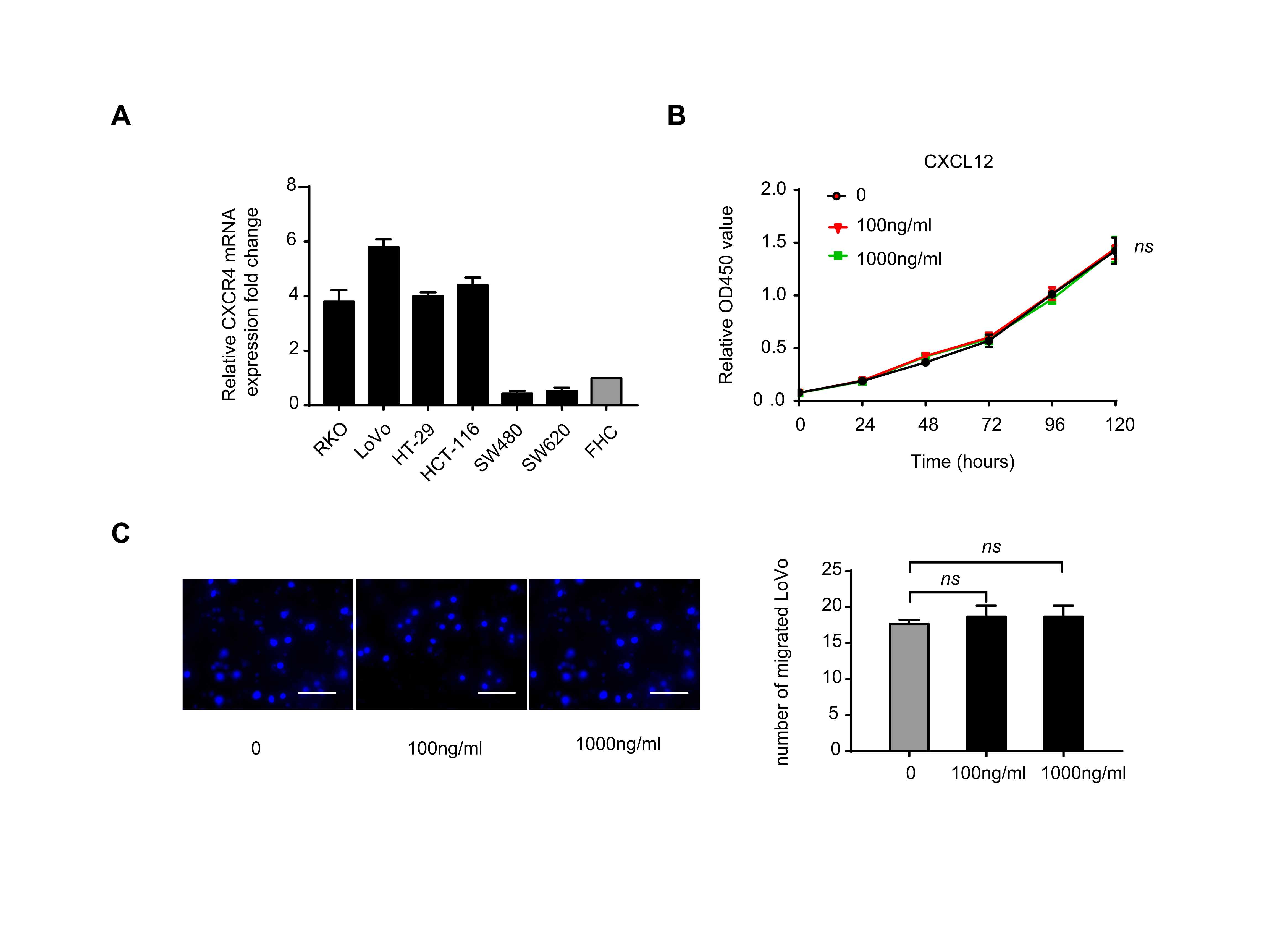

Supplement: Supplementary file 4 — Fig. S4. The effect of CXCL12 on CRC cell proliferation and migration. (A) Real‐time PCR showed that CXCR4, the receptor of CXCL12, is highly expressed in LoVo cells as compared to the other CRC cell lines. (B) Cell viability assay showed that addition of recombinant CXCL12 at 0, 100, and 1000 ng/ml final concentration did not influence the LoVo cancer cell proliferation. Cell migration was also evaluated in LoVo cells (C). Scale bars: 100 μm, 40× magnification. Error bars were represented as mean ± SD (n = 3). p‐values were analyzed with one‐way ANOVA. [file MOL2-13-2460-s004.tif]
