# Supplementary material for: Klotho‐mediated targeting of CCL2 suppresses the induction of colorectal cancer progression by stromal cell senescent microenvironments
Source: Mol Oncol. 2019 Oct 6;13(11):2460–75. doi: 10.1002/1878-0261.12577 (PMC6822285; doi:10.1002/1878-0261.12577)
Supplement: Supplementary file 5 — Table S1. The association of Klotho expression with clinicopathological features inpatients with CRC. Table S2. The senescence‐associated secretory phenotype (SASP) associated genes. [file MOL2-13-2460-s005.docx]

**Table S1**

**The association of Klotho expression with clinicopathological features in patients with CRC.**

| **Variables** | **CRC patients**  **(n=143)** | **%** | **Klotho expression（n=143）** | | ***P* value** |
| --- | --- | --- | --- | --- | --- |
|  |  |  | **Low（n=60）** | **High（n=83）** |  |
| Age |  |  |  |  | 0.036 |
| ≤70 | 79 | 55.2% | 27（45%） | 52（62.7%） |  |
| >70 | 64 | 44.8% | 33（55%） | 31（37.3%） |  |
| Gender |  |  |  |  | 0.736 |
| Male | 81 | 56.7% | 33（55%） | 48（57.8%） |  |
| Female | 62 | 43.3% | 27（45%） | 35（42.2%） |  |
| Differentiation |  |  |  |  | 0.187 |
| Well/Moderate | 90 | 62.9% | 34（56.7%） | 56（67.5%） |  |
| Poor | 52 | 37.1% | 26（43.3%） | 27（22.5%） |  |
| Lymph node metastasis |  |  |  |  | <0.01 |
| Negative | 82 | 47.3% | 23（38.3%） | 59（71.1%） |  |
| Positive | 61 | 42.7% | 37（61.7%） | 24（28.9%） |  |
| Distant metastasis |  |  |  |  | <0.01 |
| Yes | 24 | 16.8% | 19（31.7%） | 5（6.0%） |  |
| No | 119 | 73.2% | 41（68.3%） | 78（94.0%） |  |
| TNM stage |  |  |  |  | <0.01 |
| I、II | 77 | 53.8% | 14（23.3%） | 63（75.9%） |  |
| III、IV | 66 | 47.2% | 46（76.7%） | 20（24.1%） |  |

**Table S2**

**The senescence-associated secretory phenotype (SASP) associated genes.**

| **Gene Symbol** | **Gene description** | **Gene function** |
| --- | --- | --- |
| CXCL5 | C-X-C motif chemokine ligand 5 | Inflammatory response, signal transduction |
| CXCL14 | C-X-C motif chemokine ligand 14 | Immune response, cell-cell signaling, signal transduction |
| CXCL12 | C-X-C motif chemokine ligand 12 | G protein-coupled receptor signaling pathway, defense response |
| CCL2 | C-X-C motif chemokine ligand 2 | G protein-coupled receptor signaling pathway, inflammatory response |
| GM-CSF | Colony Stimulating Factor 2 | cellular response to granulocyte macrophage colony-stimulating factor stimulus, cytokine-mediated signaling pathway, |
| B2M | beta-2-microglobulin | innate immune response, cellular protein metabolic process |
| IL-8 | C-X-C Motif Chemokine Ligand 8 | G protein-coupled receptor signaling pathway, cell cycle arrest |
| CXCL1 | C-X-C Motif Chemokine Ligand 1 | G protein-coupled receptor signaling pathway, |
| RPS13 | ribosomal protein S13 | Protein biosynthesis |
| STAT3 | Signal Transducer And Activator Of Transcription 3 | Aging, acute-phase response |
| IL-1β | Interleukin 1 Beta | Cell-cell signaling, apoptotic process |
| IL-13 | Interleukin 13 | cytokine-mediated signaling pathway, immune response |
| EGFR | Epidermal Growth Factor Receptor | cell population proliferation, MAPK cascade |
| FN | Fibronectin | acute-phase response, cellular protein metabolic process |
| IL-7 | Interleukin 7 | Immune response, positive regulation of cell population proliferation |
| IGF-BP4 | Insulin Like Growth Factor Binding Protein 4 | regulation of cell growth, inflammatory response |
| TNF-α | Tumor necrosis factor alpha | I-kappaB kinase/NF-kappaB signaling, inflammatory response |
| VEGF | vascular endothelial growth factor | Angiogenesis, positive regulation of epithelial cell proliferation |
| MCP-4 | Monocyte Chemoattractant Protein 4 | G protein-coupled receptor signaling pathway, inflammatory response |
| ENA78 | Epithelial-Derived Neutrophil-Activating Peptide 78 | G protein-coupled receptor signaling pathway, cell-cell signaling |
| GRO-a | growth-related oncogene-Alpha | G protein-coupled receptor signaling pathway, immune response |
| CTSB | Cathepsin B | collagen catabolic process, regulation of apoptotic process |
| IGF-BP6 | Insulin Like Growth Factor Binding Protein 6 | regulation of insulin-like growth factor receptor signaling pathway, negative regulation of cell population proliferation |
| ANG | Angiogenin | Angiogenesis, cell migration |
| IFN-ϒ | Interferon Gamma | adaptive immune response, cell cycle arrest |
| ICAM-3 | Intercellular Adhesion Molecule 3 | Cell adhesion, cell-cell adhesion |
| IL-6 | Interleukin 7 | acute-phase response, inflammatory response |
| bFGF | Basic Fibroblast Growth Factor | MAPK cascade, cytokine-mediated signaling pathway |
| SCF | Stem Cell Factor | MAPK cascade, cell adhesion |
| tPA | Tissue-Type Plasminogen Activator | blood coagulation, cellular protein modification process |
| GRO-b | growth-related oncogene-beta | G protein-coupled receptor signaling pathway, cytokine-mediated signaling pathway |
| ICAM-1 | Intercellular Adhesion Molecule 3 | T cell antigen processing and presentation, cell aging |
| I-TAC | Interferon-Inducible T-Cell Alpha Chemoattractant | G protein-coupled receptor signaling pathway, T cell chemotaxis |
| TIMP2 | Tissue Inhibitor Of Metalloproteinases 2 | Aging, response to cytokine |
| HGF | hepatocyte  growth factor | cytokine-mediated signaling pathway, mitotic cell cycle |
| uPA | Urokinase plasminogen activator | blood coagulation, proteolysis |
| MMP3 | Matrix Metallopeptidase 3 | cytokine-mediated signaling pathway, collagen catabolic process |
| MCP-2 | Monocyte Chemoattractant Protein 2 | G protein-coupled receptor signaling pathway, cell-cell signaling |
| GRO-g | growth-related oncogene-gamma | G protein-coupled receptor signaling pathway, inflammatory response |
| Nos-2 | Nitric Oxide Synthase 2 | cytokine-mediated signaling pathway, arginine catabolic process |
| KGF | Keratinocyte Growth Factor | fibroblast growth factor receptor signaling pathway, response to wounding |
| CCL-8 | C-C Motif Chemokine Ligand 8 | G protein-coupled receptor signaling pathway, cell-cell signaling |
| PAI-2 | Plasminogen Activator Inhibitor 2 | Fibrinolysis, negative regulation of apoptotic process |
| AREG | Amphiregulin | Cell signaling, cell proliferation |
| TGF-β1 | Transforming Growth Factor Beta 1 | Aging, cell cycle arrest |
| MMP10 | Matrix Metalloproteinase 10 | collagen catabolic process, extracellular matrix organization |
| IGF-BP7 | Insulin Like Growth Factor Binding Protein 7 | Negative regulation of cell proliferation, cell growth |
| MIP-1a | Macrophage Inflammatory Protein 1-Alpha | G protein-coupled receptor signaling pathway, cytokine-mediated signaling pathway |
| TRAIL-R3 | tumor necrosis factor–related apoptosis-inducing ligand receptor 3 | TRAIL-activated apoptotic signaling pathway, regulation of apoptotic process |
| MMP1 | Matrix Metallopeptidase 1 | cellular protein metabolic process, collagen catabolic process |
| PIGF | Phosphatidylinositol Glycan Anchor Biosynthesis Class F | GPI anchor biosynthetic process, preassembly of GPI anchor in ER membrane |
| EREG | Epiregulin | cytokine-mediated signaling pathway, cell-cell signaling |
| MIP-3a | Macrophage Inflammatory Protein 3 Alpha | G protein-coupled receptor signaling pathway, T cell migration |
| PGE2 | prostaglandin E2 | Prostaglandin metabolism, Signal Transduction |
| Fas | Fas Cell Surface Death Receptor | Fas signaling pathway, apoptotic process |
| MMP14 | Matrix Metallopeptidase 14 | Angiogenesis, cell motility |
| M-CSF | Macrophage Colony-Stimulating Factor 1 | cell differentiation, cell population proliferation |
| Arg1 | Arginase 1 | Aging, adaptive immune response |
| HRG | Heregulin | Signaling transduction, cell growth |
| IGF-BP3 | Insulin Like Growth Factor Binding Protein 3 | apoptotic process, cellular protein metabolic process |
| MMP8 | Matrix Metallopeptidase 8 | collagen catabolic process, negative regulation of gene expression |
| IGF-BP2 | Insulin Like Growth Factor Binding Protein 2 | Aging, cellular protein metabolic process |
| CCL-7 | C-C Motif Chemokine Ligand 7 | G protein-coupled receptor signaling pathway, cell-cell signaling |
| OPG | osteoprotegerin | apoptotic process, extracellular matrix organization |
| MMP13 | Matrix Metallopeptidase 13 | collagen catabolic process, cellular protein metabolic process |
| COL1A1 | Collagen Type I Alpha 1 Chain | collagen biosynthetic process, collagen fibril organization |
| MIF | Macrophage Migration Inhibitory Factor | Cell aging, cell population proliferation |
| IL-15 | Interleukin 15 | Aging, NK T cell proliferation |
| IL-1α | Interleukin 1 Alpha | apoptotic process, immune response |
| HCC-4 | C-C Motif Chemokine Ligand 16 | cell communication, cell-cell signaling |
| BLC | B Lymphocyte Chemoattractant | G protein-coupled receptor signaling pathway, cell-cell signaling |
| MMP12 | Matrix Metallopeptidase 12 | collagen catabolic process, elastin catabolic process |
| IL-10 | Interleukin 10 | Aging, B cell proliferation |
| u-PAR | Urokinase plasminogen activator surface receptor | blood coagulation, negative regulation of apoptotic process |
| SGP130 | Soluble membrane Glycoprotein 130 | cytokine-mediated signaling pathway, response to cytokine |
| PAI-1 | Plasminogen Activator Inhibitor 1 | chronological cell aging, angiogenesis |
| TECK | Thymus Expressed Chemokine | G protein-coupled receptor signaling pathway, immune response |
| Eotaxin-3 | C-C Motif Chemokine Ligand 26 | G protein-coupled receptor signaling pathway, cell-cell signaling |
